# The Structural Basis of Binding Stability and Selectivity of Sarolaner Enantiomers for *Ctenocephalides felis* RDL Receptors

**DOI:** 10.3390/molecules30132756

**Published:** 2025-06-26

**Authors:** Xiaojiao Zheng, Xin Wang, Xiulian Ju, Zhichao Ma, Genyan Liu

**Affiliations:** 1 Hubei Provincial Engineering Research Center of Racing Horse Detection and Application Transformation, Equine Science Research and Horse Doping Control Laboratory, School of Physical Education, Wuhan Business University, Wuhan 430056, China; zhengxiaojiao916@163.com (X.Z.); wxzh0919@163.com (X.W.); 2 Hubei Key Laboratory of Novel Reactor and Green Chemical Technology, School of Chemical Engineering and Pharmacy, Wuhan Institute of Technology, Wuhan 430205, China; xiulianju2008@aliyun.com

**Keywords:** GABA receptor, sarolaner, molecular mechanisms, molecular docking, molecular dynamics simulations

## Abstract

The ionotropic γ-aminobutyric acid (GABA) receptor (GABAR) is a key target for the development of antiparasitic agents, particularly against ectoparasites, such as fleas and ticks. Binding stability and selectivity of sarolaner enantiomers for *Ctenocephalides felis* RDL receptors (RDLR) were investigated in the current study. Wild-type (WT) *C. felis* RDLR and its A285S mutant were constructed using homology-based, fragment-based threading and AI-driven approaches, of which, SWISS-MODEL generated the most reliable structures. Molecular docking showed that the sarolaner *S*-enantiomer had higher binding affinity for both receptors than the *R*-enantiomer, primarily due to hydrogen bonding with Ile256, π–π stacking with Phe326, and hydrophobic interactions with Ile267 and Ile268. Molecular dynamics simulations confirmed the binding stability of the *S*-enantiomer-receptor complex in which key residues maintained interactions throughout the trajectories. Binding free energy analysis supported these results and highlighted the role of nonpolar interactions in binding stability. The A285S mutation had minimal impact on the binding pocket, and the *S*-enantiomer remained selective for and bound to the mutant receptor. Insights into the insecticidal mechanism of sarolaner enantiomers are given, and the current findings may inform the development of veterinary drugs from novel isoxazoline-based NAMs targeting insect GABARs.

## 1. Introduction

The ionotropic γ-aminobutyric acid (GABA) receptor (GABAR) is a member of the pentameric ligand-gated ion channel (pLGIC) superfamily. It is characterized by Cys-loop topology [1] and is composed of five homologous and heterologous subunits assembled into a chloride (Cl^−^) channel [2]. GABAR binding to the endogenous neurotransmitter, GABA, causes rapid neuronal inhibition in the central nervous system (CNS) and spinal cord in both mammalian and insect brains [3].

**Figure 1:**
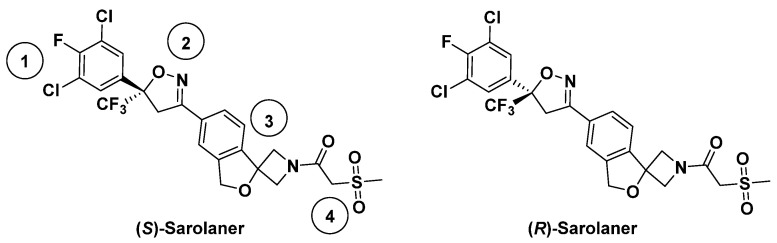
Chemical structures of sarolaner enantiomer. Take the *S*-enantiomer for example. (1) Phenyl head group; (2) isoxazoline core; (3) spiroazetidinebenzofuran moiety; (4) methylsulfonylethanone tail.

Insect GABARs differ from their mammalian counterparts due to the lack of the A/B/C subunit classification [4]. Mammalian ionotropic GABA_A_Rs have five subunits composed of 19 isotypes [5,6], but insect GABARs are either homo-pentameric with “*resistant-to-dieldrin*” (RDL) subunits or hetero-pentameric with some unidentified subunits [7,8]. Only the RDL subunit has been shown to form functional homomeric GABA-gated chloride (Cl^−^) channels with pharmacological properties similar to native insect GABARs [9], and insect GABARs are often referred to as RDL GABA receptors (RDLRs). Insect RDLRs share structural similarities with pLGICs, each subunit having an extracellular β-fold domain (ECD), four transmembrane α-helices (TM1–TM4), and a flexible intracellular loop [10]. Miller and Aricescu resolved the crystal structure of a homopentameric human GABA_A_R composed of β3 subunits in 2014, facilitating the study of mammalian and insect GABARs [11]. Insect RDLRs are targets of insecticides and antiparasitic agents [12]. Ectoparasites, such as fleas, ticks, and mites, cause systemic and localized disease in companion animals and contribute to zoonotic disease transmission [13]. Parasiticides are available for the ectoparasite protection of dogs and cats, but innovative veterinary products that are effective, safe, and convenient are required. Isoxazoline GABAR negative allosteric modulators (NAMs) are a novel class of veterinary drugs, in which mechanisms of action have been studied [14]. Isoxazolines have been shown to exert antiparasitic effects by selective blockade of insect GABA- and glutamate-gated ion channels, showing higher sensitivity for the former than for the latter [15]. Previous studies have focused on agrochemical development [16,17], but the potential for targeting insect RDLR for animal health applications has received recent attention [18,19]. Sarolaner, afoxolaner, fluralaner, and lotilaner are the new generation isooxazoline-based parasiticides [20,21,22,23] with functions as insecticides and acaricides but are considered safe for vertebrates [14]. Sarolaner is an isoxazoline-azetidine compound developed for ectoparasite control in companion animals and is highly effective in preventing reinfestation by fleas, ticks, and mites [24]. The sarolaner structure has four components: a substituted phenyl ring (head group), an isoxazoline core, a spiroazetidinebenzofuran moiety, and a methylsulfonylethane tail (Figure 1). It is synthesized exclusively as the biologically active *S*-enantiomer since the *R*-enantiomer lacks antiparasitic activity [25]. While no published data directly comparing the bioactivity of sarolaner enantiomers are currently available, the decision to develop the S-enantiomer alone suggests a significant difference in potency. Similar enantioselectivity has been reported for related isoxazoline compounds, such as lotilaner, where in vitro assays demonstrated that the *S-*enantiomer was 10–100 times more potent than the *R*-enantiomer against *C*. *felis* and *Rhipicephalus sanguineus* [20,26]. These findings support a class-wide trend favoring the *S*-enantiomer as the bioactive isomer. Sarolaner is distinguished from other isoxazolines by having a spiroazetidinebenzofuran moiety, giving structural rigidity and functional efficacy. The structure has not been previously reported. Sarolaner has been found to have up to 99% acaricidal efficacy against four tick species: *Rhipicephalus sanguineus*, *Dermacentor reticulatus*, *Ixodes Ricinus*, and *Amblyomma americanum* [27].

The sarolaner *S*-enantiomer has insecticidal activity against both wild-type (WT) *C. felis* RDLR and the resistant A285S mutant [25]. However, mechanisms underlying binding and selectivity remain poorly understood. WT and A285S mutant receptors were modeled in the current study, and sarolaner enantiomer binding was characterized using molecular docking, MOLCAD protein surface analysis, and molecular dynamics (MD) simulations. The aim was to clarify the molecular mechanism of enantiomer selectivity and inform the design of safe and effective insect GABAR-targeting parasiticides.

## 2. Results

### 2.1. Prediction and Validation of Receptor Structures

Three-dimensional structures of wild-type (WT) *C. felis* RDLR and its resistant A285S mutant were constructed using three prediction tools: template-based homology modeling (SWISS-MODEL) [28], fragment-based threading (I-TASSER) [29], and AI-driven de novo structure prediction (AlphaFold 2.0) [30]. Homology modeling relies on > 30% sequence similarity between target and template to enable successful alignment [31]. Sequence alignment showed that the human GABA_A_R β3 subunit has 47.8% sequence identity with the WT *C. felis* RDL subunit and 47.4% with the A285S mutant. Alignment of target sequences and template protein shows a high degree of similarity, particularly within the TM domain (Figures S1 and S2, red regions represent identical amino acid sequences). The SWISS-MODEL structures were likely to be the most accurate. I-TASSER and AlphaFold 2.0 generated receptor structures based only on target protein sequences.

**Figure 2:**
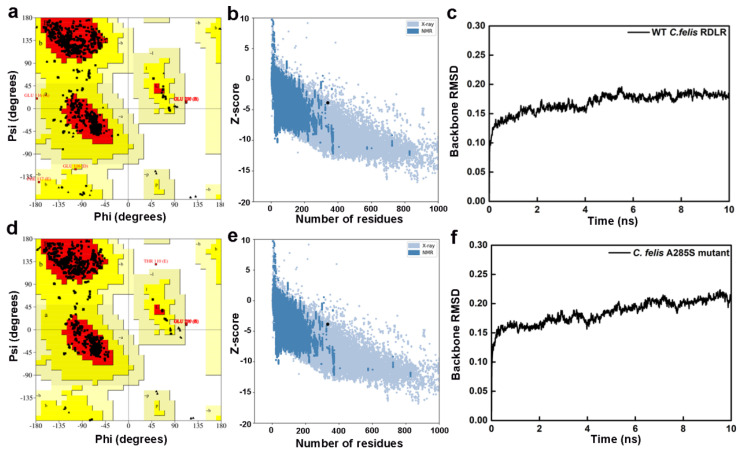
Evaluation results of the constructed WT *C. felis* RDLR and the A285S mutant homology models. Ramachandran plots of the WT *C. felis* RDLR (**a**) and the mutant (**d**) models; Z-score distribution plots of the WT *C. felis* RDLR (**b**) and the mutant (**e**) models; RMSD plots of backbone atoms of the WT *C. felis* RDLR (**c**) and the mutant (**f**) models during the 10 ns MD simulation.

Receptor structure accuracy was evaluated by PROCHECK and ProSA workspace (Table 1) [32,33]. Ramachandran plots of WT *C. felis* RDLR modeled by SWISS-MODEL showed 93.5% distribution in the most favored regions (red), 6.0% in additional allowed regions (yellow), 0.2% in generously allowed regions (light yellow), and 0.3% in disallowed regions (white) (Figure 2a). Plots of the A285S mutant showed respective distributions of 94.2%, 5.4%, 0.0%, and 0.4% (Figure 2d). Z-scores were −3.86 for WT and −3.85 for mutant receptors, which fall within the typical range observed for database natural proteins of comparable size (represented by dark blue and light blue dots, Figure 2b,e). The WT *C. felis* RDLR and A285S mutant homology models were considered reliable and suitable for further analysis. The SWISS-MODEL had superior performance across various parameters in predicting *C. felis* RDLRs, compared with I-TASSER and AlphaFold 2.0, due to the high sequence identity between target amino acid sequences and template receptor and to rational adjustments applied to target sequences based on the template. In addition, internal evaluation metrics from the SWISS-MODEL server supported the structural quality of the WT and A285S models. GMQE values were 0.69 and 0.70, and QMEANDisCo scores were 0.73 and 0.72, indicating reliable global and local accuracy, especially within the transmembrane binding site. All key ligand-interacting residues were located in high-confidence regions (Figure S3, Table S1) [34].

I-TASSER and AlphaFold 2.0 receptor models had more amino acid residues in disallowed regions compared with SWISS-MODEL (Figures S3 and S4). I-TASSER Ramachandran plots had < 80% residues located in core regions, which undermined the structural rationality of these regions in the *C. felis* RDLR I-TASSER models. In addition, intracellular big loop regions, consisting of complex disordered regions involving free movement of amino acid residues, predicted by AlphaFold 2.0 showed low prediction confidence. Disordered regions remain a challenge for structural biologists. AlphaFold 2.0 is an advanced tool that integrates cutting-edge AI algorithms and excels in predicting orphan proteins with limited information on homologous structures. However, homology modeling is more accurate for target sequences with high sequence identity to reliable template proteins. In conclusion, the SWISS-MODEL online server produced the most appropriate *C. felis* RDLR models for subsequent analysis.

**Figure 3:**
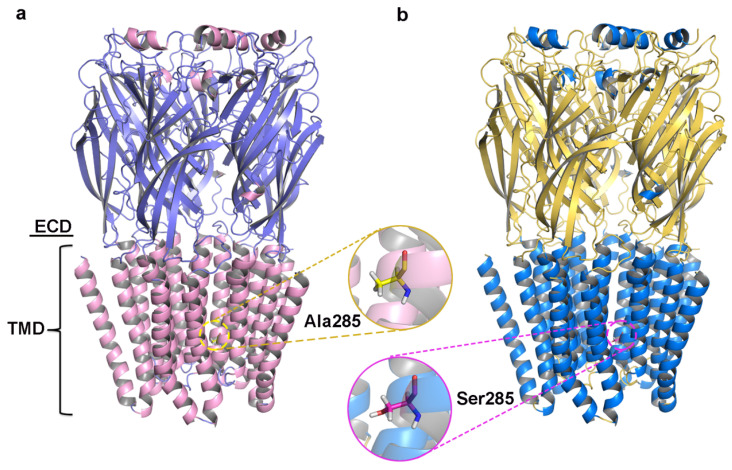
The structures of *C. felis* RDLR models optimized by the 10 ns MD simulation; the front view of the receptor exhibits the different domains: the ECD and the TMD. (**a**) WT *C. felis* RDLR: α-helixes are depicted in pink, β-sheets shown in purple, and Ala285 residue in TM2 is represented by the yellow stick. (**b**) *C. felis* A285S mutant: α-helixes are depicted in marine, β-sheets shown in yellow, and Ser285 residue in TM2 is indicated by the magenta stick.

Initial models were optimized with a 10 ns MD simulation and potential energy convergence profiles for WT and A285S mutant *C. felis* RDLR are shown in Figure S5. System energy reached convergence at 4230 ps for the WT receptor and 3673 ps for the A285S mutant. Optimization was monitored by root mean square deviation (RMSD) of backbone atoms relative to initial structures (Figure 2c,f). WT *C. felis* RDLR stabilized and converged to an RMSD value of 0.17 nm after 6 ns. Minor fluctuations were observed at 4 ns for the A285S mutant, after which stabilization occurred. Both receptor protein systems approached convergence with the correction of residues in disallowed regions, and a stable model structure was achieved. Structural optimization of three-dimensional models of MD-optimized WT and A285S mutant *C. felis* RDLR was considered complete (Figure 3).

### 2.2. Molecular Docking

**Figure 4:**
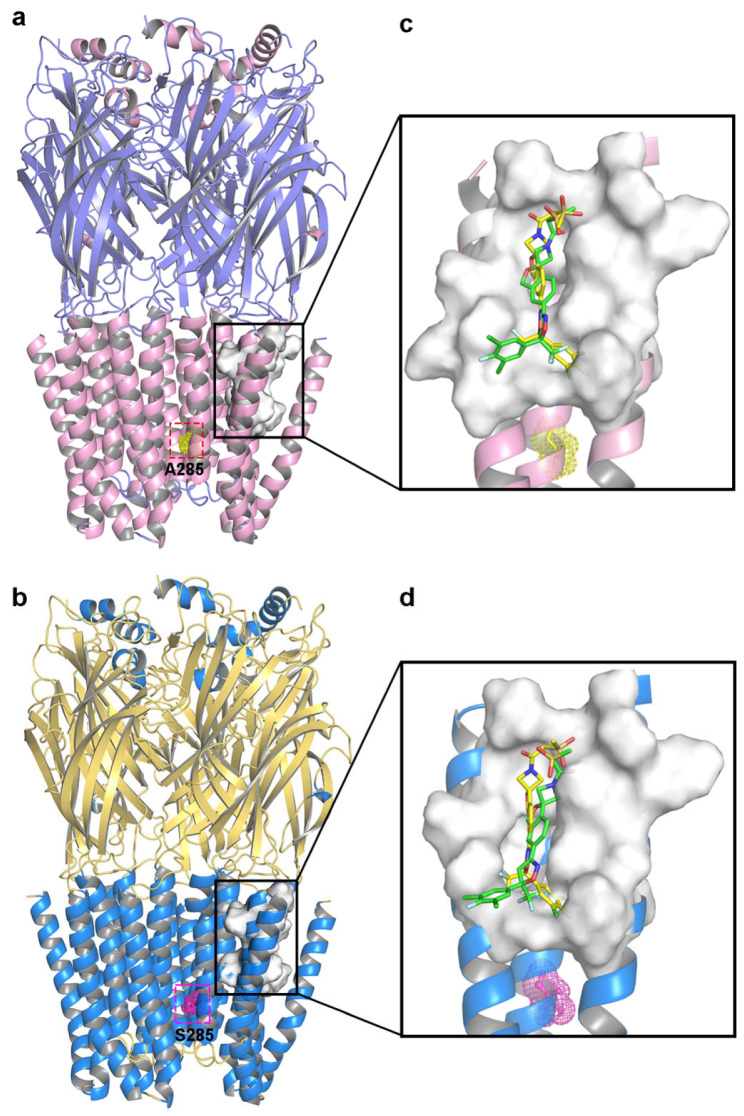
Representations of the binding pocket of *C. felis* RDLRs. (**a**) WT *C. felis* RDLR: Ala285 residue of TM2 is depicted in yellow mesh; (**b**) *C. felis* A285S mutant: Ser285 residue in TM2 is indicated by magenta mesh. The surfaces of the binding pocket of WT *C. felis* RDLR (**c**) and the A285S mutant (**d**) with sarolaner enantiomers in it (*S*-enantiomer: yellow; *R*-enantiomer: green).

Molecular docking allowed the docking pockets of *C. felis* WT and A285S mutant RDLRs bound to sarolaner enantiomers to be investigated using the Surflex-Dock geom mode (Figure 4). The secondary structures of receptor proteins are color-coded, and binding pocket surfaces are highlighted in white. Ala285 of the WT RDLR TM2 region was located near the inner channel, separate from the sarolaner binding pocket, which may explain why the resistant A285S mutation does not affect sarolaner docking (Table 2). Total_Score docking values for the sarolaner *S*-enantiomer were 5.35 with a binding energy of −30.54 kJ/mol for WT and 5.18 with a binding energy of −29.54 kJ/mol for the A285S mutant, which is similar but higher than for the *R*-enantiomer. The ranking of Total_Score values aligns well with inhibitory potency.

**Figure 5:**
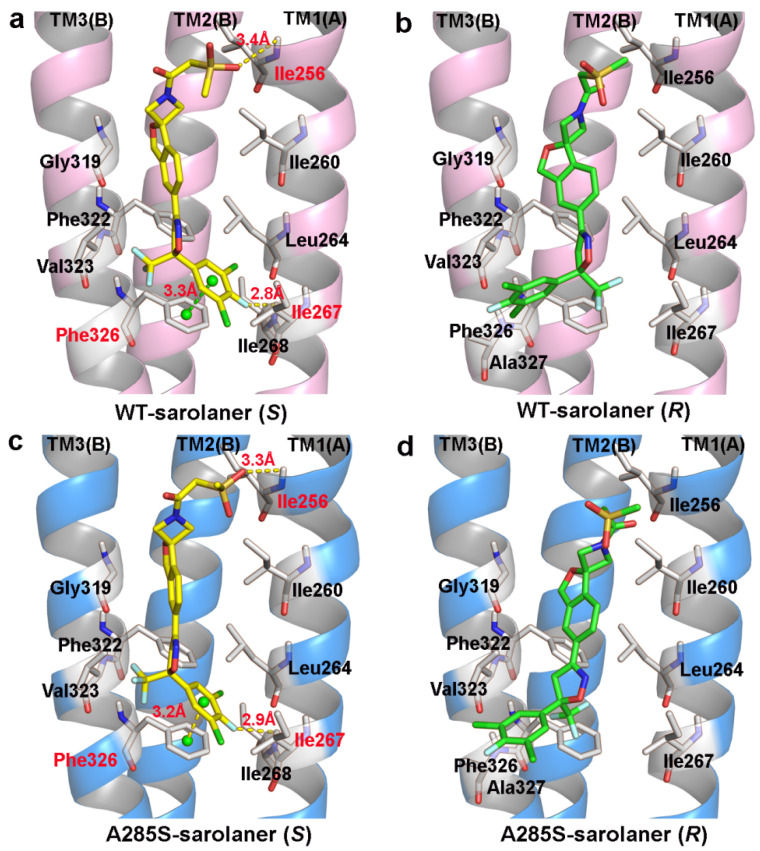
Binding pattern of the two sarolaner enantiomers in binding pocket of *C. felis* WT-RDLR and its A285S mutant. (**a**,**c**) Binding mode of sarolaner *S*-enantiomer in the binding site of the WT *C. felis* RDLR and A285S mutant; (**b**,**d**) Binding mode of sarolaner *R*-enantiomer in the binding pocket of the WT *C. felis* RDLR and A285S mutant. (WT-RDLR: pink cartoon; A285S mutant: marine cartoon; *S*-enantiomer: yellow stick; *R*-enantiomer: green stick). Important residues are labeled with residue identifiers and numbers, shown as white sticks. The hydrogen bonds and π-π stacking distances are shown as yellow and green dashes with their lengths (Å), respectively.

Binding modes of the sarolaner *S*-enantiomer were similar for WT RDLR and the A285S mutant (Figure 5). Key interactions are labeled directly in the 3D views to provide spatial clarity, and although 2D diagrams are not included, the current representations comprehensively capture the key interaction features. As shown in Figure 5a, the sarolaner *S*-enantiomer spiroazetidinebenzofuran scaffold contributes to molecular rigidity and is inserted in a perpendicular orientation into the WT RDLR binding pocket. A key π-π stacking interaction was identified between the phenyl ring and Phe326 (TM3), with an intermolecular distance of 3.3 Å. The fluorine substituent of the phenyl ring engaged in strong hydrogen bonding with Ile267 (TM1) (Ile267-N-H…F-C, bond length: 2.8 Å), and another hydrogen bond was observed between the methylsulfinyl tail group and Ile256 (TM1) (Ile256-N-H…O, bond length: 3.4 Å). The phenyl ring head group was deeply embedded within a large hydrophobic pocket formed by Leu264, Ile267, and Ile268 (TM1), which stabilized *S*-enantiomer binding to WT *C. felis* RDLR. The A285S mutant showed similar π-π stacking interactions, hydrogen bonds, and hydrophobic effects (Figure 5c). Differences were observed in the interaction of the *R*-enantiomer with WT and A285S mutant *C. felis* RDLR (Figure 4b,d). The *R*-enantiomer had a stereochemical configuration which caused 180° inversion of the phenyl ring, causing the phenyl ring head group to form weak hydrophobic interactions with TM3 Val323 and Phe326 within the binding site. The *R*-enantiomer did not embed deeply within the binding pocket, leading to the loss of hydrogen bonding and associated non-covalent interactions. These differences may explain the lack of *R*-enantiomer-inhibitory activity against WT and the A285S mutant RDLR.

In conclusion, TM1 residues; Ile256, Ile267, and Ile268; and the adjacent TM3 Phe326 stabilize the interaction between the sarolaner *S*-enantiomer and *C. felis* RDLRs. The size and intrinsic properties of the binding pocket do not appear to be substantially altered by the A285S mutation.

### 2.3. Computational Analysis of MOLCAD Protein Surface

**Figure 6:**
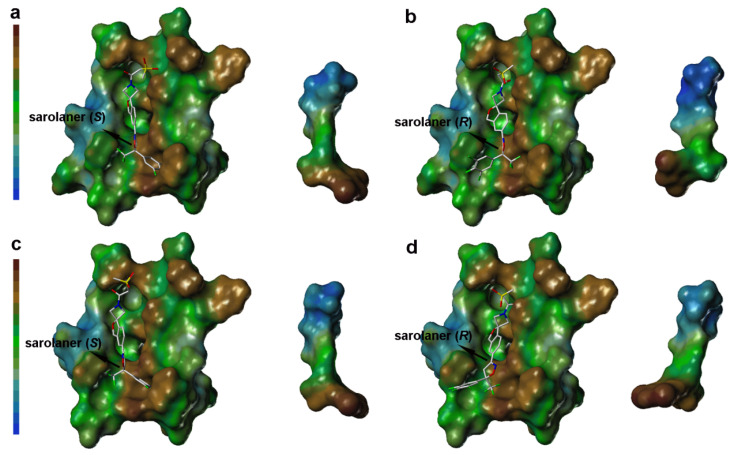
Surface diagram of lipophilic potential of sarolaner enantiomers and the binding pocket of WT *C. felis* RDLR and its A285S mutant. (**a**) Lipotropy potential surface of sarolaner (*S*) and the binding pocket of WT *C. felis* RDLR protein; (**b**) Lipotropy potential surface of sarolaner (*R*) and the binding pocket of WT RDLR protein; (**c**) Lipotropy potential surface of sarolaner (*S*) and the binding pocket of A285S mutant; (**d**) Lipotropy potential surface of sarolaner (*R*) and the binding pocket of A285S mutant.

Lipophilic potential (LP) surface properties for the *C. felis* RDLR and A285S mutant binding pockets are shown in Figure 6. The *S*-enantiomer phenyl ring head group and binding site residues are shown with a brown surface, indicating favorable hydrophobic interactions for ligand binding (Figure 6a), and hydrophobic interactions stabilize binding to both WT and A285S mutant RDLR (Figure 6c). The *S*-enantiomer isoxazoline core did not show significant lipophilicity or hydrophilicity. The LP surface of the spiroazetidinebenzofuran moiety transitioned from green to light blue, reflecting hydrophilic potential, and the adjacent binding site amino acid residues also had hydrophilic LP surface properties, suggesting that hydrophilic groups may contribute to binding affinity in this part of the binding pocket.

**Figure 7:**
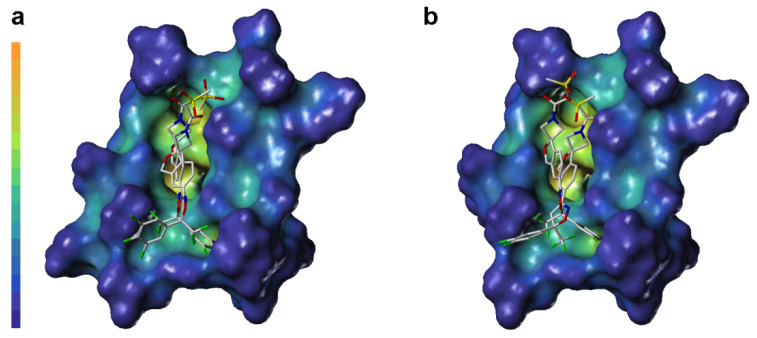
Surface diagram of cavity depth of sarolaner enantiomers and the binding pocket of WT *C. felis* RDLR (**a**) and A285S mutant (**b**).

LP surface properties of the sarolaner methylsulfonylethane tail group showed hydrophilic potential, in contrast with its lipophilic binding site residues. This may induce a torsional shift in the sulfonyl group, increasing solvent exposure and maximizing the ligand pharmacokinetic properties. Mutation of Ala285 to Ser285 did not change the LP surface properties of the binding pocket, consistent with previous docking analysis and LP surface profile (Figure 6c). By contrast, the *R*-enantiomer phenyl ring head group had hydrophobic surface properties which made weak interactions with the neutral LP surface of the adjacent binding pocket residues (Figure 6b). The altered ligand configuration resulted in the loss of hydrophobic interactions on the right side of the binding pocket, preventing deep embedding of the *R*-enantiomer and impairing the stability of the ligand-receptor complex (Figure 6d). Cavity depths (CDs) for *R*- and *S-*enantiomers with WT and A285S mutant *C. felis* RDLR are shown in Figure 7. Binding pocket surface areas were 2246.88 Å^2^ with a CD of 5.9961 Å for WT and 2211.59 Å^2^ with a CD of 6.1124 Å for A285S mutant RDLR, indicating minimal differences and suggesting little impact of the mutation on the sarolaner binding pocket. Thus, the above molecular docking conformations are shown to be rational and useful for the design of isoxazoline compounds with different stereochemical configurations.

### 2.4. Molecular Dynamics Simulation

**Figure 8:**
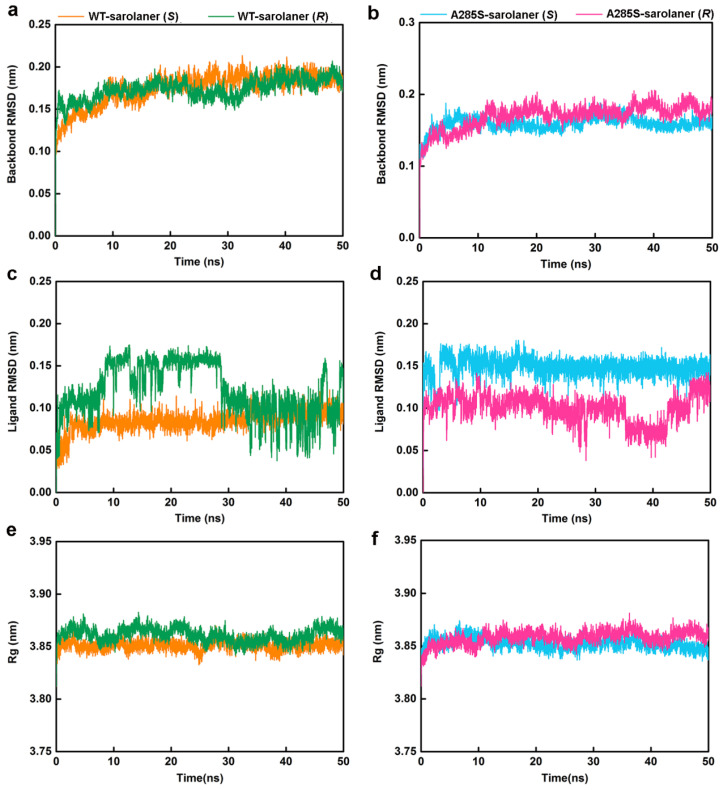
MD simulation results of four protein-ligand complexes. WT-sarolaner (*S*) (orange), WT-sarolaner (*R*) (green), A285S-sarolaner (*S*) (azure), and A285S-sarolaner (*R*) (pink). (**a**,**b**) RMSDs of the *C. felis* RDLR and A285S-RDLR mutant backbone atoms; (**c**,**d**) RMSDs of ligands; (**e**,**f**) Rg values of backbone atoms.

**Figure 9:**
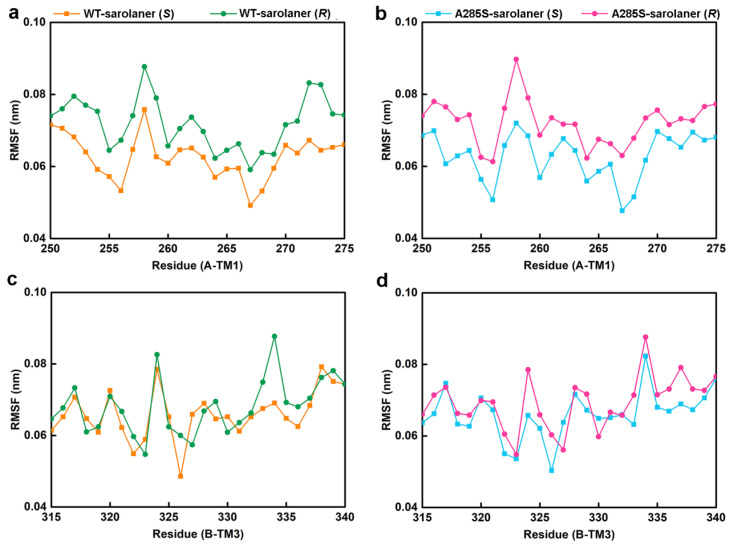
The residue RMSF results of the four complexes. (**a**) C-α RMSF values of residues in the TM1 domain of WT-sarolaner (*S*) system (orange) based on the full trajectory; (**b**) C-α RMSF values of residues in the TM3 domain of A285S-sarolaner (*S*) system (azure); (**c**) C-α RMSF values of residues in the TM1 domain of WT-sarolaner (*R*) system (green); (**d**) C-α RMSF values of residues in the TM3 domain of A285S-sarolaner (*R*) system (pink).

MD simulations were conducted at 50 ns on four systems: WT-sarolaner (*S*) (orange), WT-sarolaner (*R*) (green), A285S-sarolaner (*S*) (azure), and A285S-sarolaner (*R*) (pink). Dynamic parameters of RMSD (backbone and ligand), root mean square fluctuation (RMSF), and radius of gyration (Rg) were monitored to evaluate trajectory changes (Figure 8 and Figure 9).

RMSD analysis offers insights into protein-ligand complex stability [35]. Backbone RMSD values of WT-sarolaner (*S*) and A285S-sarolaner (*S*) complexes stabilized after 10 ns, converging at 0.18 nm (WT) and 0.16 nm (mutant), but WT-sarolaner (*R*) and A285S-sarolaner (*R*) complexes showed greater fluctuation in RMSD throughout the simulation, suggesting higher conformational variability compared to the *S*-enantiomer complexes. The reduced stability of complexes containing the *R*-enantiomer may be due to weaker hydrophobic interactions or steric clashes. RMSD values of sarolaner (*S*)-enantiomer complexes converged after 10 ns, showing consistent ranges of 0.07~0.10 nm (WT) and 0.14~0.16 nm (mutant, Figure 8c,d), but those of *R*-enantiomer complexes showed greater fluctuations throughout the MD trajectory. RMSD trajectories of both protein backbones and ligands indicate the stability of sarolaner (*S*)-enantiomer complexes with WT and A285S mutant *C. felis* RDLR, whereas sarolaner (*R*)-enantiomer complexes are much less stable. Rg trajectories were examined to evaluate compactness and protein backbone conformational changes during MD simulations. Rg values of backbone Cα atoms in the WT-sarolaner (*S*) and A285S-sarolaner (*S*) complexes remained stable throughout the 50 ns MD simulation, achieving equilibrium at relatively low Rg levels and maintaining values around 3.85 nm (Figure 8e,f). These findings indicate that the binding of sarolaner (*S*)-enantiomer enhanced the compactness of the receptor backbone structure during the MD simulations. By contrast, binding of the sarolaner (*R*)-enantiomer resulted in slight fluctuations of Rg trajectories of the receptor’s Cα atoms, consistent with the RMSD results for receptor backbone atoms. Thus, the sarolaner *S*-enantiomer stabilizes the protein backbone, resulting in enhanced stability of *C. felis* RDLR-ligand complexes, compared with *R*-enantiomer complexes.

RMSF analysis indicates the flexibility of individual amino acid residues in a protein chain, giving insight into the structural flexibility of the whole protein. The TM1 domain of chain A and the TM3 domain of chain B, the location of the receptor-ligand binding pocket, were analyzed (Figure 9). RMSF trajectories gave similar fluctuation amplitudes for most binding pocket residues across all four *C. felis* RDLR-sarolaner complexes, suggesting a similar binding mode for WT and mutant RDLRs. RMSF values were 0.047 to 0.091 nm for binding pocket residues, indicating low flexibility, and were generally lower in the WT-sarolaner (*S*) complex than in the WT-sarolaner (*R*) complex (Figure 8a,c). The *S*-enantiomer may maintain a more stable binding environment within the pocket than the *R*-enantiomer, and Ile267 (TM1) and Ile268 (TM3) had particularly low RMSF values, reflecting their role in stabilization. In addition, Ile256 in TM1 of chain A and Phe326 in TM3 of chain B also had low RMSF values and a stabilization role. Key amino acid residues with low flexibility in all four complexes may thus be responsible for the stability of the sarolaner (*S)*-enantiomer binding pocket interaction. Hydrophobic contacts, hydrogen bonds, and π-π stacking interactions were likely to be involved. Key residues with low flexibility were also observed in the A285S-sarolaner (*S*) complex and RMSF values were lower than for the A285S-sarolaner (*R*) complex (Figure 9b), suggesting more stable interactions within the A285S-sarolaner (*S*) complex. RMSF analysis thus explains some stability differences between the WT and mutant *C. felis* RDLR with the two enantiomers. Hydrophobic interactions, hydrogen bonding, and π-π stacking interactions between the sarolaner (*S*)-enantiomer and the *C. felis* RDLR binding pocket are seen to be crucial for stable binding and may account for the high selectivity of the ligand.

The initial, middle, and final binding conformations of the *R*- and *S*-enantiomers with both WT and A285S mutant RDLRs were extracted at 0 ns, 25 ns, and 50 ns, respectively. As shown in Figure S7, the *S*-enantiomer maintained a relatively stable conformation throughout the simulation in the WT RDLR, with only minor structural adjustments. Similarly, in the A285S mutant system (Figure S8), the *S*-enantiomer preserved its binding orientation over time. In contrast, the *R*-enantiomer underwent noticeable conformational shifts during the 50 ns simulation, particularly in the mutant system, indicating weaker binding stability compared to the *S*-enantiomer.

While MD simulations in this study were performed in explicit water without a lipid bilayer, this approach is widely used in insect GABA receptor-ligand studies [31,36,37], offering an efficient and reliable framework for analyzing binding affinity and stereoselectivity. Future work will consider incorporating membrane models to enhance physiological relevance.

### 2.5. Binding Free Energy Analysis

Binding energies were calculated and analyzed by Poisson–Boltzmann surface area (MM-PBSA) molecular mechanics [38] (Table 3). Binding free energy (Δ*G*_binding_) values were −140.9 kJ/mol for WT-sarolaner (*S*), −108.5 kJ/mol for WT-sarolaner (*R*), −134.9 kJ/mol for A285S-sarolaner (*S*), and −99.5 kJ/mol for A285S-sarolaner (*R*). Δ*G*_binding_ values were stronger for complexes of the *S*-enantiomer with both WT and A285S mutant RDLR than with the *R*-enantiomer, consistent with the formation of more stable interactions by the *S*-enantiomer. Molecular docking Total_Score rankings indicated that van der Waals energy dominated binding energy with similar SASA contributions. The unfavorable polar solvation energy in complexes of WT and A285S with the (*R*) enantiomer partially offset the favorable van der Waals contributions, resulting in weaker binding. The *S*-enantiomer-WT complex showed a van der Waals energy contribution of −216.5 kJ/mol and an *S*-enantiomer-A285S complex of −219.5 kJ/mol, indicating the contribution of nonpolar interactions to stability. In summary, binding free energy substantiates the superior affinity and stability of the *S*-enantiomer in complexes with RDLR. While the A285S-sarolaner (*R*) complex exhibited a more favorable electrostatic energy (Δ*E*_ele_ = −47.9 kJ/mol), this was offset by the highest polar solvation penalty (+ 159.5 kJ/mol), resulting in a weaker net binding free energy. This suggests that electrostatic gains may be masked by desolvation effects, and that van der Waals interactions are the more consistent contributors to binding stability in these complexes. In summary, binding free energy substantiates the superior affinity and stability of the *S*-enantiomer in complexes with RDLR.

**Figure 10:**
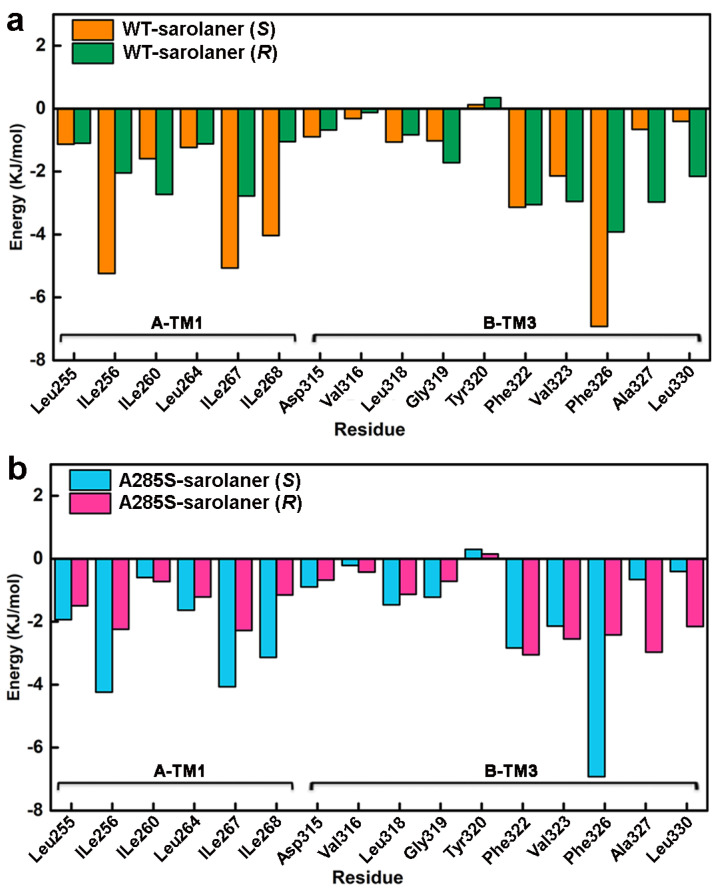
Residue-based decomposition energy plot during MD simulations. (**a**) Energy contribution values of key residues near the binding pocket of WT-sarolaner (*S*) (orange) and WT-sarolaner (*R*) (green) complexes; (**b**) Energy contribution values of key amino acids around the binding sites of A285S-sarolaner (*S*) (azure) and A285S-sarolane (*R*) (pink) complexes.

Energy decomposition analysis was performed to identify key amino acid residues involved in complex formation (Figure 10), and those with interaction energies <−3.00 kJ/mol were considered to have a positive stabilizing effect [39]. Docking results showed that TM1 and adjacent TM3 residues in the binding pocket, including Ile256, Ile260, Leu264, Ile267, Ile268, Gly319, Leu318, Phe322, Phe323, Phe326, and Phe327, engaged in hydrogen bonding, hydrophobic interactions, and other nonpolar interactions with sarolaner enantiomers, contributing to stability. Phe326 of the TM3 domain showed the highest contribution of −6.9214 kJ/mol to the WT-*S*-enantiomer complex, highlighting the role of π-π stacking interactions. TM1 residues, Ile267 and Ile268, also made significant contributions of −5.0651 kJ/mol and −4.0343 kJ/mol, respectively, which were higher than for the same residues in the WT-sarolaner (*R*) complex (−2.3758 kJ/mol and −1.0475 kJ/mol). TM1 Ile256 also made a more favorable binding contribution (−5.2391 kJ/mol) to the *S*-enantiomer complex than to the *R*-enantiomer. Ile267 and Ile268 made favorable hydrophobic interactions with the phenyl group of the *S*-enantiomer, and Ile256 (TM1) formed hydrogen bonds with the *S*-enantiomer oxygen atom. Such interactions were not observed with the *R*-enantiomer (Figure 10b). In conclusion, nonpolar residues, Ile267 (TM1), Ile268 (TM1), Phe326 (TM3), and Ile256 (TM1), are key amino acids responsible for stable binding of the *S*-enantiomer to the WT and A285S mutant *C. felis* RDLR, consistent with the docking analysis and RMSF results described earlier.

## 3. Materials and Methods

### 3.1. Protein Structure Prediction

*C. felis* is a model organism for antiparasitic research. Little crystallographic data was available, and three-dimensional structures of WT and A285S mutant RDLR were predicted using SWISS-MODEL, I-TASSER, and AlphaFold 2.0 [28,29,30] with the most reliable receptor model selected for subsequent experiments.

Protein sequences for *C. felis* RDL subunit (UniProt ID: V9Z984) and resistant mutant S285-RDL subunit (UniProt ID: V9ZCF2), each with 483 amino acids, were retrieved from UniProtKB database (http://www.uniprot.org), and the human β3 GABA_A_R crystal structure (PDB code: 4COF, 2.96 Å) was chosen as a template for homology modeling, as in previous studies of the insect RDLR [31,40]. The structure is available at high resolution and has structural homology as a pentameric receptor [11]. The crystal structure of 4COF was retrieved from the RCSB Protein Data Bank (https://www.rcsb.org). The Cys-loop receptor family incorporates a complex intracellular loop region that is unresolved, and the large intracellular loop between the TM3 and TM4 transmembrane domains of the template protein was replaced with a short peptide (S-Q-P-A-R-A-A) to optimize structural stability and monodispersity [31]. A similar adjustment was made to residues 341~440 of the target sequences to ensure the rationality of subunit construction. Sequence alignments were performed using the Clustal-W online server (https://www.genome.jp/tools-bin/clustalw, accessed on 5 April 2024) [41], comparing *C. felis* WT and S285-RDL target sequences with the 4COF template, and alignments were visualized with Espript 3.0 (http://espript.ibcp.fr/ESPript/cgi-bin/ESPript.cgi, accessed on 10 April 2024) [42]. Structural models were generated with the SWISS-MODEL User Template module (https://swissmodel.expasy.org/interactive, accessed on 1 May 2024). FASTA files for the two target sequences were submitted to I-TASSER (https://zhanggroup.org/I-TASSER/, accessed on 10 May 2024) and AlphaFold 2.0 (https://www.alphafold.ebi.ac.uk/, accessed on 20 May 2024) for structure prediction. *C. felis* RDLR is a homologous pentameric transmembrane protein, allowing individual subunit chains to be modeled separately and assembled into the complete pentameric structure.

The three structural models were evaluated by SAVES v6.0 (https://saves.mbi.ucla.edu/, accessed on 2 June 2024) [32] and ProSA-web (https://prosa.services.came.sbg.ac.at/prosa.php, accessed on 10 June 2024) [33], and the model with the highest score was selected for a 10 ns MD simulation using GROMACS 2019.5 software, yielding a stable and rational protein model.

### 3.2. Ligand Docking

Receptor–ligand interactions were explored at the atomic level by molecular docking [43]. Sarolaner *R*- and *S*-enantiomers were constructed with the Sketch module in SYBYL-X 2.1, and ligand energy minimization was performed by Tripos force field with Gasteiger–Hückel charges, a gradient convergence criterion of 0.0005 kcal/(mol·Å), and a maximum of 10,000 iterations. All other parameters were set to default values to achieve stable molecular energy states [44]. Molecular docking was performed in Surflex-Dock Geom mode to enhance docking precision [45]. The WT and A285S mutant *C. felis* RDLR 3D structures were derived from homology models, and a semi-flexible docking approach in Residue mode was chosen to generate the docking protomol [40]. The binding pocket was defined within a 5 Å radius of key residues located at the interface of adjacent subunits: TM1 residues Ile260, Leu264, Ile267, and Val271 and TM3 residues, Gly319, Val323, Phe322, and Phe326. This binding site selection is supported by prior studies highlighting the critical role of these TM1 and TM3 interface residues in mediating isoxazoline binding and conferring resistance through point mutations in insect RDRs [15,46,47]. The conformation with the highest total score was selected for binding mode characterization and MD simulations. Molecular docking visualizations were generated using PyMOL 2.3.3 (DeLano Scientific LLC, San Carlos, CA, USA) [48].

### 3.3. Receptor Protein Surface Calculation

The MOLCAD module integrated in SYBYL-X 2.1 was used to visualize receptor surface properties and illustrate the physicochemical properties of the protein and ligand interactions [49]. The protein surface was generated in Fast Connolly mode, and binding pocket LP and CD were calculated after docking. Protein and ligand surfaces were visualized with color gradients, with deep brown representing highly lipophilic regions and blue indicating strongly hydrophilic areas. Hydrophobic interactions were highlighted.

### 3.4. Molecular Dynamics Simulations

MD simulations were conducted at 50 ns on protein-ligand complexes using GROMACS 2019.5 software (Uppsala University, Stockholm University and the Royal Institute of Technology, Sweden) on a Linux-based platform [50,51]. Protein topological files were generated with the pdb2gmx module, and ligand topological parameters were added by the ACPYPE web tool [52]. As sarolaner lacks unusual atoms or highly polarizable groups, the generated parameters were deemed suitable. No missing parameters were reported, and the topology files were visually verified. Subsequently, complexes were solvated in the SPC/E water model with a buffer distance of about 12 Å between protein and cubic box edges to facilitate protein diffusion simulation [53]. Twenty chloride ions were added to neutralize system charge.

Energy minimization was conducted using steepest descent and conjugate gradient methods until van der Waals contact energies dropped below 10 kJ/mol. NVT equilibrium was carried out at 1 bar and NPT equilibrium at 300 K to stabilize the system. MD simulations at 50 ns with 2 fs per step were performed. Complex stability and conformational change were analyzed by RMSD, RMSF, and Rg [39]. Binding free energy (∆*G*_binding_) was calculated using the MM-PBSA method [38], and the g_mmpbsa module was used to extract the final 5 ns of the equilibrium trajectory during MD simulation [54]. ∆*G*_binding_ was calculated as the difference in free energy between the complex (*G*_complex_) and the sum of the free energies of the protein (*G*_free-protein_) and the ligand (*G*_free-ligand_), as shown in the following Formula (1):

(1) $$∆G_{binding}=G_{complex}-{(G}_{free-protein}+G_{free-ligand})$$

The free energy associated with each state was calculated as follows (2):

(2) $$G=E_{MM}+G_{sol}-TS$$

where *E*_MM_ and *G*_sol_ represent the molecular mechanics average potential energy in a vacuum and under solvation, respectively, as defined by Equations (3) and (4). TS denotes the entropy contribution to the free energy in a vacuum.

(3) $$E_{MM}=E_{bonded}+E_{nonbonded}=E_{bonded}+E_{ele}+E_{vdW}$$

(4) $$G_{sol}=G_{polar}+G_{nonpolar}$$

where *E*_MM_ is composed of bonding energy (*E*_bond_) and non-bonding energy (*E*_nonbonded_) interactions. Electrostatic (*E*_ele_) and van der Waals (*E*_vdW_) interactions together constitute the *E*_nonbonded_, and *G*_sol_ is the sum of polar solvation energy (*G*_polar_) and non-polar solvation energy (*G*_nonpolar_). The built-in MmPbSaStat.py program was used to extract the binding energies and quantify the contribution of key residues to total binding free energy. In this study, entropy contributions were not included in the MM-PBSA calculations, consistent with widely accepted end-point free energy protocols. As our primary objective was to compare the binding strength and stability between *R-* and *S*-enantiomers, enthalpy-based estimates are considered sufficient for drawing reliable mechanistic conclusions [55,56].

## 4. Conclusions

Binding modes and selectivity of sarolaner *R-* and *S-*enantiomers for *C. felis* RDLR were investigated. The *S*-enantiomer bound WT and A285S mutant RDLR more tightly than the *R*-enantiomer. Structural models and MD simulations demonstrated stable complexes between the *S*-enantiomer and WT and A285S mutant *C. felis* RDLR. Residues in TM1, Ile256, Ile267, Ile268, TM3, and Phe326 stabilized the interaction between the sarolaner *S*-enantiomer and both receptors. Hydrophobic interactions, hydrogen bonds, and π-π stacking interactions determined the high selectivity and binding stability of the *S*-enantiomer. Our findings contribute insights into the actions of isoxazoline-based NAMs and may be used to inform the design of novel antiparasitic agents targeting insect GABARs. Further studies incorporating both open and closed channel conformations, together with in vivo validation and structural optimization of sarolaner derivatives, may promote the development of ectoparasiticides with improved potency and selectivity.

## Figures and Tables

**Table 1:** The evaluation results of the predicted WT *C. felis* RDLR and the A285S mutant models by the three tools using the Ramachandran plot and Z-score distribution plot.

Method	Model	Proportion of the Residues in Different Regions (%)				Z-Score
		Most Favored Regions	Additional Allowed Regions	Generously Allowed Regions	Disallowed Allowed Regions	
Swiss-Model	WT-RDLR	93.5	6.0	0.2	0.3	−3.86
	A285S-RDLR	94.2	5.4	0.0	0.4	−3.85
I-TASSER	WT-RDLR	73.2	19.1	5.7	2.1	−3.47
	A285S-RDLR	77.6	16.6	4.4	1.5	−3.78
AlphaFold2	WT-RDLR	83.7	11.0	3.6	1.6	−4.22
	A285S-RDLR	86.0	9.0	2.6	2.4	−4.05

**Table 2:** Docking results of the sarolaner enantiomers in the WT *C. felis* RDLR and the A285S mutant binding pockets.

Protein-Ligand	Total_Score	Binding Energy (kJ/mol)	Intermolecular Interactions
WT-Sarolaner (*S*)	5.35	−30.54	2H-bond: Ile256, Ile267 1π-π stacking: Phe326
WT-Sarolaner (*R*)	3.42	−19.52	-
A285S-Sarolaner (*S*)	5.18	−29.54	2H-bond: Ile256, Ile267 1π-π stacking: Phe326
A285S-Sarolaner (*R*)	3.08	−17.57	-

**Table 3:** The free binding energies of the sarolaner enantiomers in the WT *C. felis* RDLR versus A285S mutant active sites.

Complex	Δ*E*_vdW_ (kJ/mol)	Δ*E*_ele_ (kJ/mol)	Δ*G*_PB_ (kJ/mol)	Δ*G*_SA_ (kJ/mol)	Δ*G*_binding_ (kJ/mol)
WT-sarolaner (*S*)	−216.5 ± 9.4	−30.6 ± 10.3	123.9 ± 14.1	−17.8 ± 0.8	−140.9 ± 13.1
WT-sarolaner (*R*)	−202.9 ± 13.0	−35.1 ± 9.2	151.0 ± 21.2	−21.4 ± 0.9	−108.5 ± 12.9
A285S-sarolaner (*S*)	−219.2 ± 134	−33.1 ± 9.9	138.1 ± 10.6	−20.7 ± 1.0	−134.9 ± 12.1
A285S-sarolaner (*R*)	−191.6 ± 16.0	−47.9 ± 11.7	159.5 ± 20.4	−19.5 ± 0.9	−99.5 ± 16.1

## Data Availability

Data is contained within the article and Supplementary Materials.

## Supplementary Materials

The following are available online at https://www.mdpi.com/article/10.3390/molecules30132756/s1. Table S1: SWISS-MODEL internal quality evaluation parameters for the WT and A285S mutant RDLR homology models. Figures S1–S2: Sequence alignment of the *C. felis* WT-RDLR model and A285S mutant model. Figure S3: Local quality estimates (QMEANDisCo) for the SWISS-MODEL-generated *C. felis* RDLR structures. Figures S4–S5: Evaluation results of Ramachandran plots and Z-scores for predicted structure models from AlphaFold2 and I-TASSER online servers. Figure S6: Potential energy of *C. felis* WT-RDLR and A285S mutant models during energy minimization.
