# Supplementary material for: The Structural Basis of Binding Stability and Selectivity of Sarolaner Enantiomers for Ctenocephalides felis RDL Receptors
Source: Molecules. 2025 Jun 26;30(13):2756. doi: 10.3390/molecules30132756 (PMC12250689; doi:10.3390/molecules30132756)
Supplement: Supplementary file 1 [file molecules-30-02756-s001.zip › molecules-3711772-supplementary.pdf]

Supporting Information

The Structural Basis of Binding Stability and Selectivity of Sarolaner Enantiomers for *Ctenocephalides felis* RDL Receptors

SUPPORTING INFORMATION

Table S1. SWISS-MODEL internal quality evaluation parameters for the WT and A285S mutant RDLR homology models

Model	GMQE	QMEANDisCo global
WT *C. felis* RDLR	0.69	0.73 ± 0.05
C. felis A285S mutant	0.70	0.72 ± 0.05

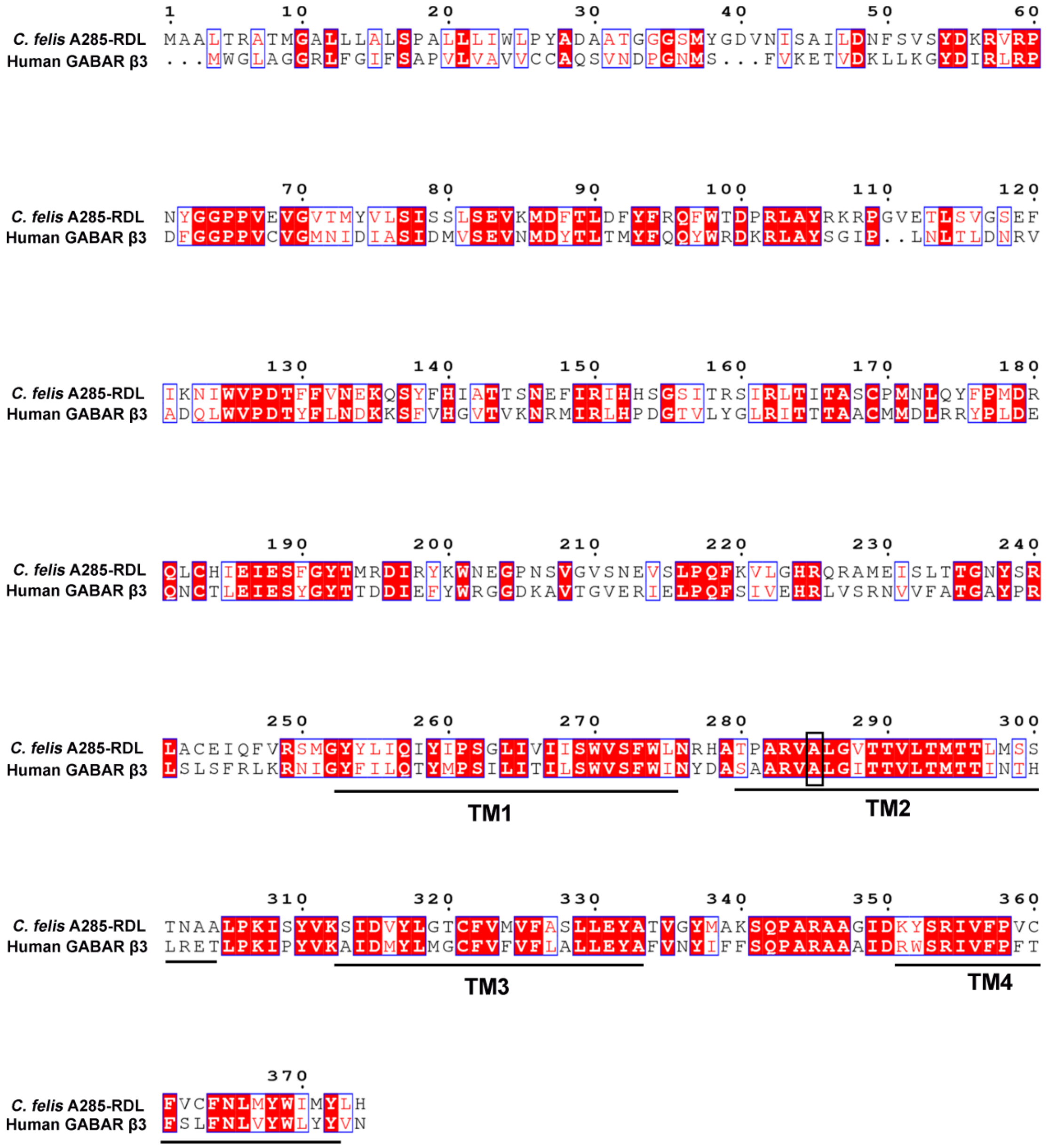

Figure S1. Sequence alignment of the human $GABA_A$ β3 subunit and the WT *C. felis* A285-RDL subunit. The mutation site are highlighted by black squares.

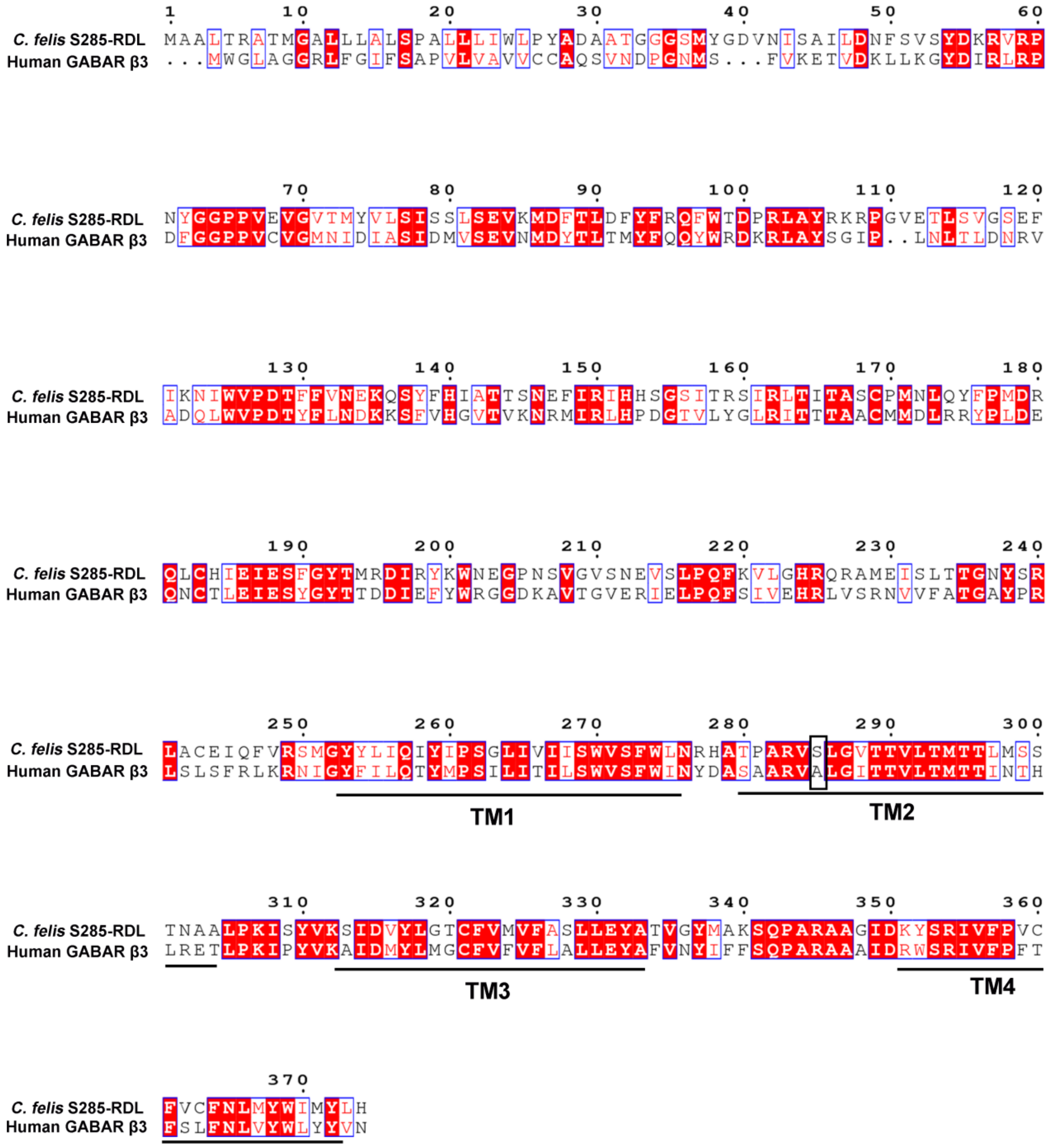

Figure S2. Sequence alignment of the human GABA$_A$ β3 subunit and the *C. felis* A285S-RDL subunit. The mutation sites are highlighted by black squares.

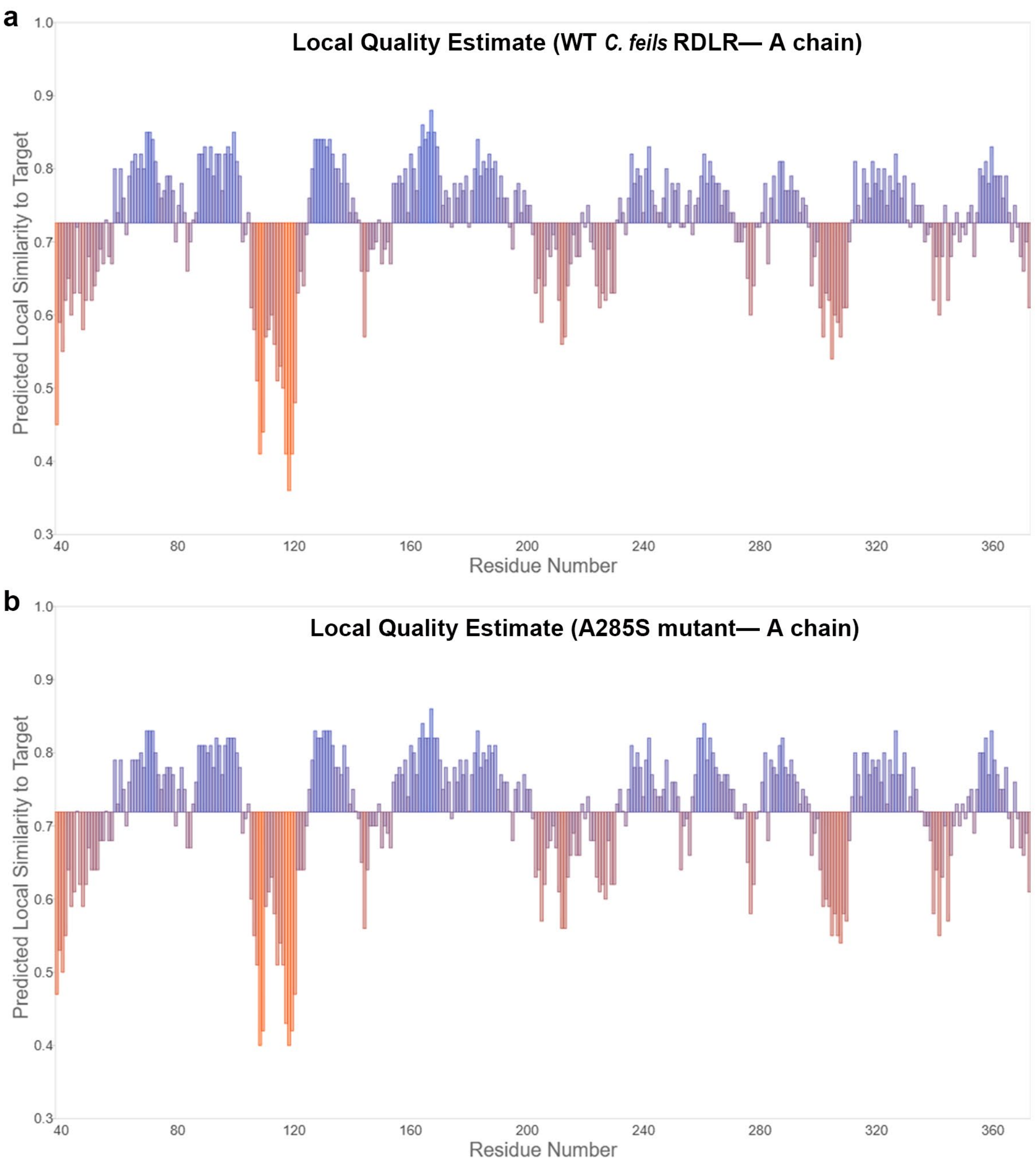

Figure S3. Local quality estimates (QMEANDisCo scores) of SWISS-MODEL-predicted *C. felis* RDLR structures. (a) WT *C. feli*s RDLR model; (b) A285S mutant model. Chain A is shown as a representative example.

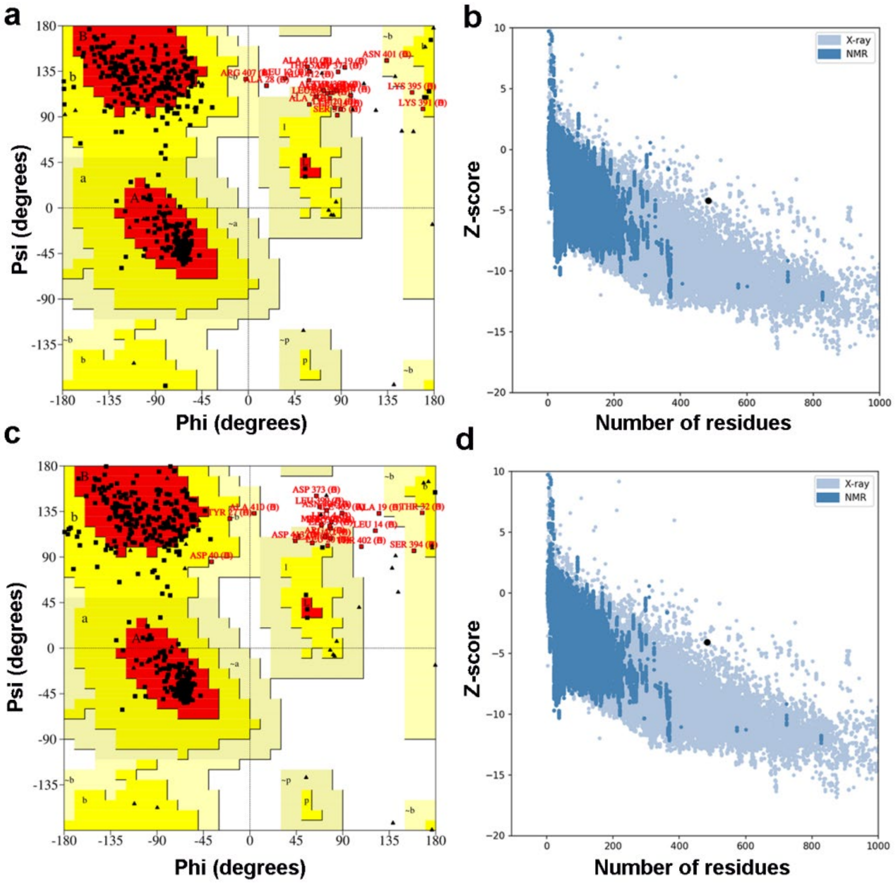

Figure S4. The evaluation results of Ramachandran (a, c) and Z-Score plot (b, d) of the predicted structure by AlphaFold2 online server. (a, b) *C. felis* WT-RDLR model; (c, d) *C. felis* A285S-RDLR model.

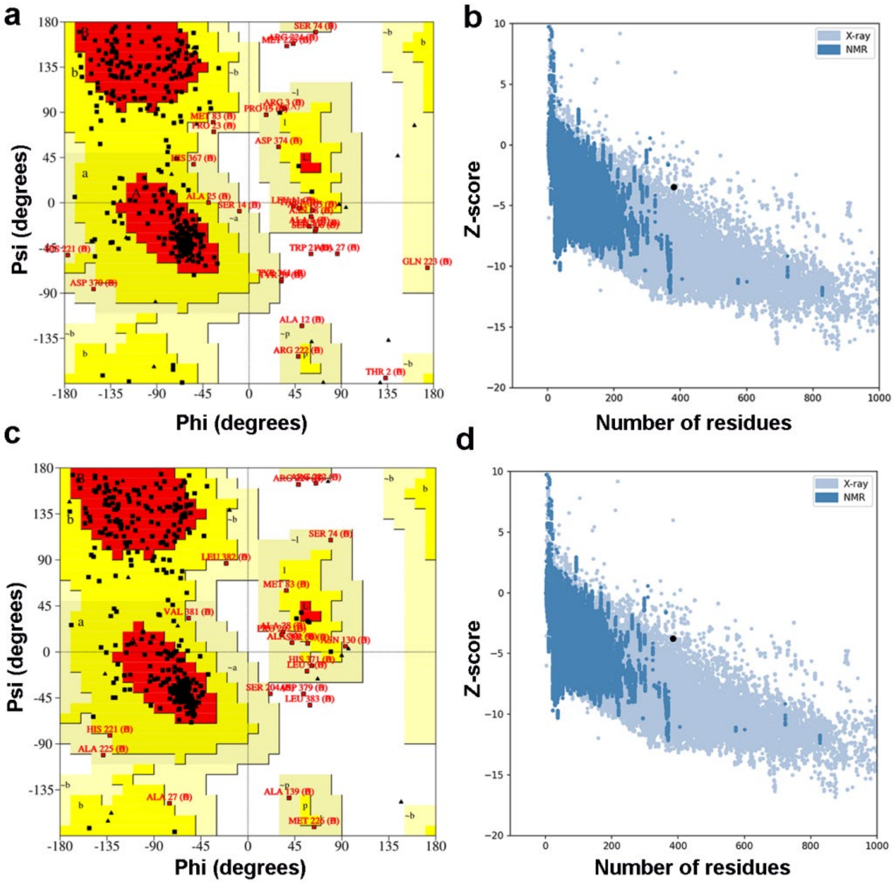

Figure S5. The evaluation results of Ramachandran (a, c) and Z-Score plot (b, d) of the predicted structure by I-TASSER online server. (a, b) *C. felis* WT-RDLR; (c, d) *C. felis* A285S-RDLR

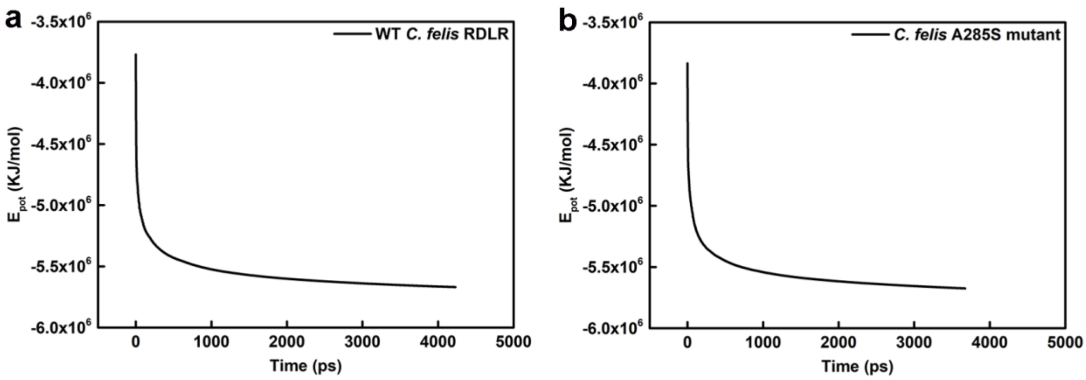

Figure S6. Potential energy of *C. felis* WT-RDLR (a) and the A285S-RDLR mutant (b) models in the step of energy minimization.

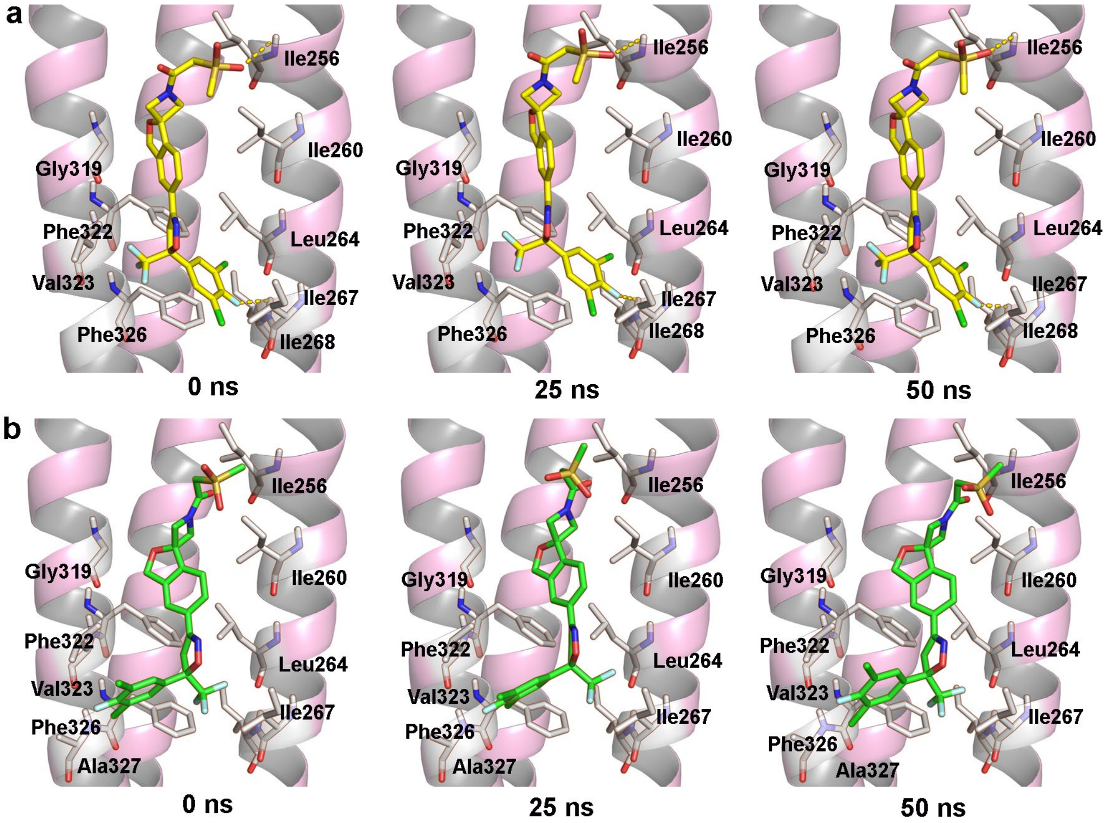

Figure S7. The initial (0 ns), middle (25 ns), and final (50 ns) binding conformations of the two sarolaner enantiomers bound to WT *C. felis* RDLR in 50 ns MD simulations, (*S*-enantiomer: yellow stick; *R*-enantiomer: green stick).

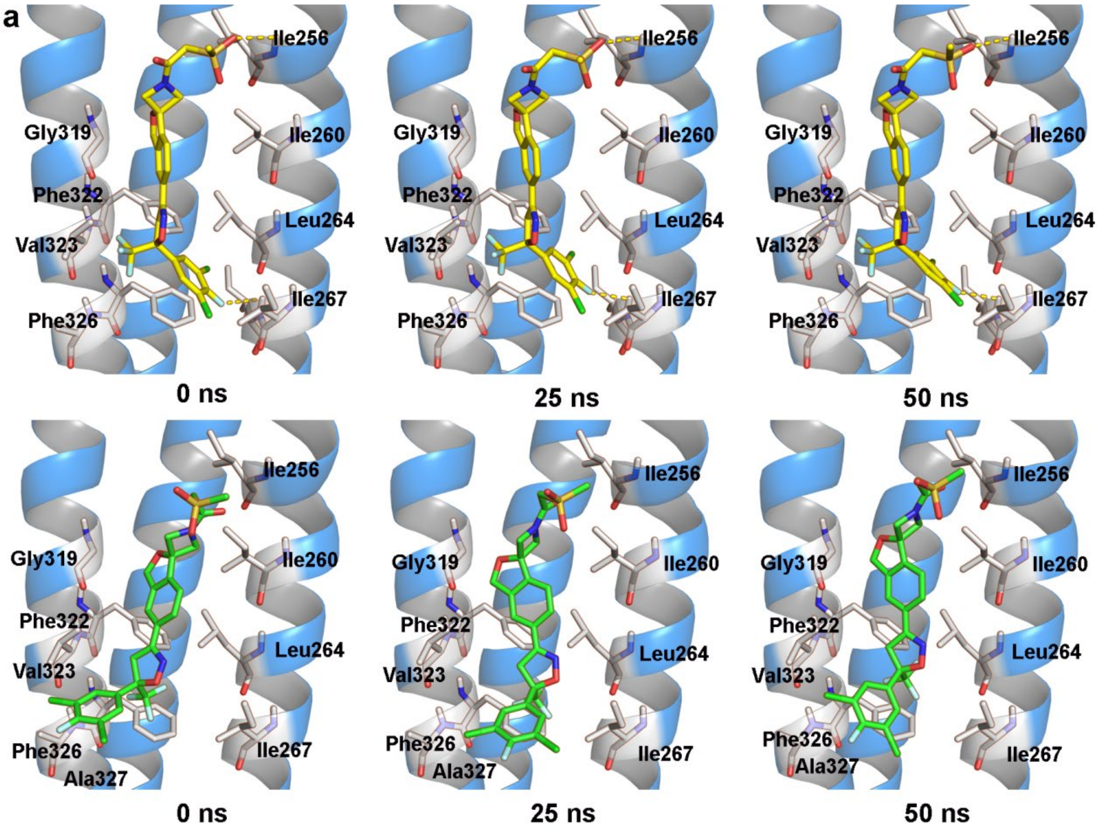

Figure S8. The initial (0 ns), middle (25 ns), and final (50 ns) binding conformations of the two sarolaner enantiomers bound to *C. felis* A285S mutant in 50 ns MD simulations, (*S*-enantiomer: yellow stick; *R*-enantiomer: green stick).
